# Why brain-controlled neuroprosthetics matter: mechanisms underlying electrical stimulation of muscles and nerves in rehabilitation

**DOI:** 10.1186/s12938-020-00824-w

**Published:** 2020-11-04

**Authors:** Matija Milosevic, Cesar Marquez-Chin, Kei Masani, Masayuki Hirata, Taishin Nomura, Milos R. Popovic, Kimitaka Nakazawa

**Affiliations:** 1 Graduate School of Engineering Science, Department of Mechanical Science and Bioengineering, Osaka University, 1-3 Machikaneyama-cho, Toyonaka, Osaka, 560-8531 Japan; 2 Institute of Biomedical Engineering, University of Toronto, 164 College Street, Toronto, ON M5S 3G9 Canada; 3 KITE Research Institute, Toronto Rehabilitation Institute - University Health Network, 520 Sutherland Drive, Toronto, ON M4G 3V9 Canada; 4 CRANIA, University Health Network & University of Toronto, 550 University Avenue, Toronto, ON M5G 2A2 Canada; 5 Department of Neurological Diagnosis and Restoration, Graduate School of Medicine, Osaka University, 2-2 Yamadaoka, Suita, Osaka 565-0871 Japan; 6 Department of Life Sciences, Graduate School of Arts and Sciences, University of Tokyo, 3-8-1 Komaba, Meguro, Tokyo, 153-8902 Japan

**Keywords:** Brain-computer interface (BCI), Functional electrical stimulation (FES), FES therapy (FEST), Hebbian plasticity, Neuroplasticity, Rehabilitation

## Abstract

Delivering short trains of electric pulses to the muscles and nerves can elicit action potentials resulting in muscle contractions. When the stimulations are sequenced to generate functional movements, such as grasping or walking, the application is referred to as functional electrical stimulation (FES). Implications of the motor and sensory recruitment of muscles using FES go beyond simple contraction of muscles. Evidence suggests that FES can induce short- and long-term neurophysiological changes in the central nervous system by varying the stimulation parameters and delivery methods. By taking advantage of this, FES has been used to restore voluntary movement in individuals with neurological injuries with a technique called FES therapy (FEST). However, long-lasting cortical re-organization (neuroplasticity) depends on the ability to synchronize the descending (voluntary) commands and the successful execution of the intended task using a FES. Brain-computer interface (BCI) technologies offer a way to synchronize cortical commands and movements generated by FES, which can be advantageous for inducing neuroplasticity. Therefore, the aim of this review paper is to discuss the neurophysiological mechanisms of electrical stimulation of muscles and nerves and how BCI-controlled FES can be used in rehabilitation to improve motor function.

## Background

Electrical stimulation can be used to deliver short electric impulses to individual or groups of muscles to cause action potentials under the stimulating electrodes, consequently producing muscle twitches and limb movements. Clinical applications of electrical stimulation first appeared in the 20th century (e.g., [68, 75, 84], to name a few). With significant developments of stimulation technology and electronic circuits, safe applications of electrical stimulation of muscles can now be used to generate controlled limb movements in individuals who have sustained central nervous system (CNS) injuries such as spinal cord injury (SCI) or stroke [116].

Individuals with CNS injuries are unable to generate and/or transmit voluntary motor commands to their muscles, resulting in reduced ability to control their limbs. This paralysis can affect their ability to produce functional movements such as reaching and grasping. Neuromuscular electrical stimulation (NMES) is a technique that can be used to activate muscles artificially and produce individual joint movements when voluntary control is affected due to CNS injury. When electrical stimulation is patterned and temporally sequenced to generate coordinated limb movements, this is referred to as functional electrical stimulation (FES). Overall, FES systems fall into the category of motor neuroprostheses [119], which are devices that use electrical stimulation to activate paralyzed muscles in a functional manner to generate limb movements [118]. While motor neuroprosthesis could include spinal cord stimulation and deep brain stimulation systems, this review will primarily focus on FES devices for stimulation of muscles and nerves. Clinical use of FES neuroprostheses includes, but is not limited to, restoration of upper and lower extremity functions, bladder and bowel functions, and respiratory function [108, 119]. Typically, FES neuroprostheses were designed to be worn as permanent assistive devices, which an individual can use to perform otherwise impaired functional movements. Such application is refereed to as prosthetic use. However, in recent years, evidence has demonstrated that application of FES over a period of time could help individuals with neurological impairments regain some of the voluntary function. By taking advantage of this therapeutic effect, FES has been used to restore voluntary upper-limb movements in individuals with neurological injuries using FES therapy (FEST) [59, 116, 144]. It should be noted that subjects are asked to attempt each movement during FEST, while FES is applied by the therapist to assist movement completion. Such associative interventions, that combine cortical activations and peripheral stimulation, likely involve Hebbian learning principles [52] to induce experience-dependant cortical re-organization (neuroplasticity) within the CNS.

Recent developments of non-invasive brain recording and processing [95] have impacted the expansion of brain-computer interface (BCI) technologies [152]. While invasive BCI-FES applications can facilitate restoration of movements [2, 22], non-invasive applications can be used for improving motor function through rehabilitation. Indeed, applications of BCI for improving motor function through rehabilitation are fast emerging [20, 30, 72]. Specifically, BCI systems translate brain signals into novel outputs, which can also be used to effectively synchronize cortical commands and movements generated by FES. Synchronized activations of cortical and peripheral networks may also facilitate associative Hebbian learning. Indeed, recent applications in rehabilitation of CNS injuries are starting to show convincing evidence of cortical neuroplasticity and improved motor function after use of BCI-controlled FES [17, 34, 58, 66, 74, 80, 93, 107]. However, despite evidence supporting recovery of voluntary function after FEST and BCI-controlled FES, little is known about the changes that occur in the CNS during and after electrical stimulation of muscles and nerves and why synchronization of cortical and muscle activations through BCI may be relevant in rehabilitation. Therefore, the objectives of this review paper are to: (A) introduce the underlying basis for generating muscle contractions using FES (Sect. “Electrical stimulation of muscles and nerves”); (B) summarize the underlying therapeutic and neurophysiological effects resulting from therapeutic application of FES (Sect. “Effects underlying electrical stimulation of muscles and nerves”); and (C) discuss the mechanisms of associative stimulation of muscles and nerves through application of BCI-controlled FES in rehabilitation (Sect. “Brain-controlled electrical stimulation of muscles and nerves in rehabilitation”). Specifically, the focus of this review will be to provide the underlying mechanisms and implications for the development of rehabilitation technologies using BCI-controlled FES to improve upper-limb voluntary motor function.

## Electrical stimulation of muscles and nerves

### Delivery of electrical stimulation

Electrical stimulation can be delivered in multiple ways, including transcutaneous and subcutaneous systems. Subcutaneous systems are typically used for applications such as bladder voiding and hand function. Such systems are be able to target the muscles more precisely and generally should require lower stimulation intensities, while invasive procedures could be more prone to infections [108].

**Fig. 1:**
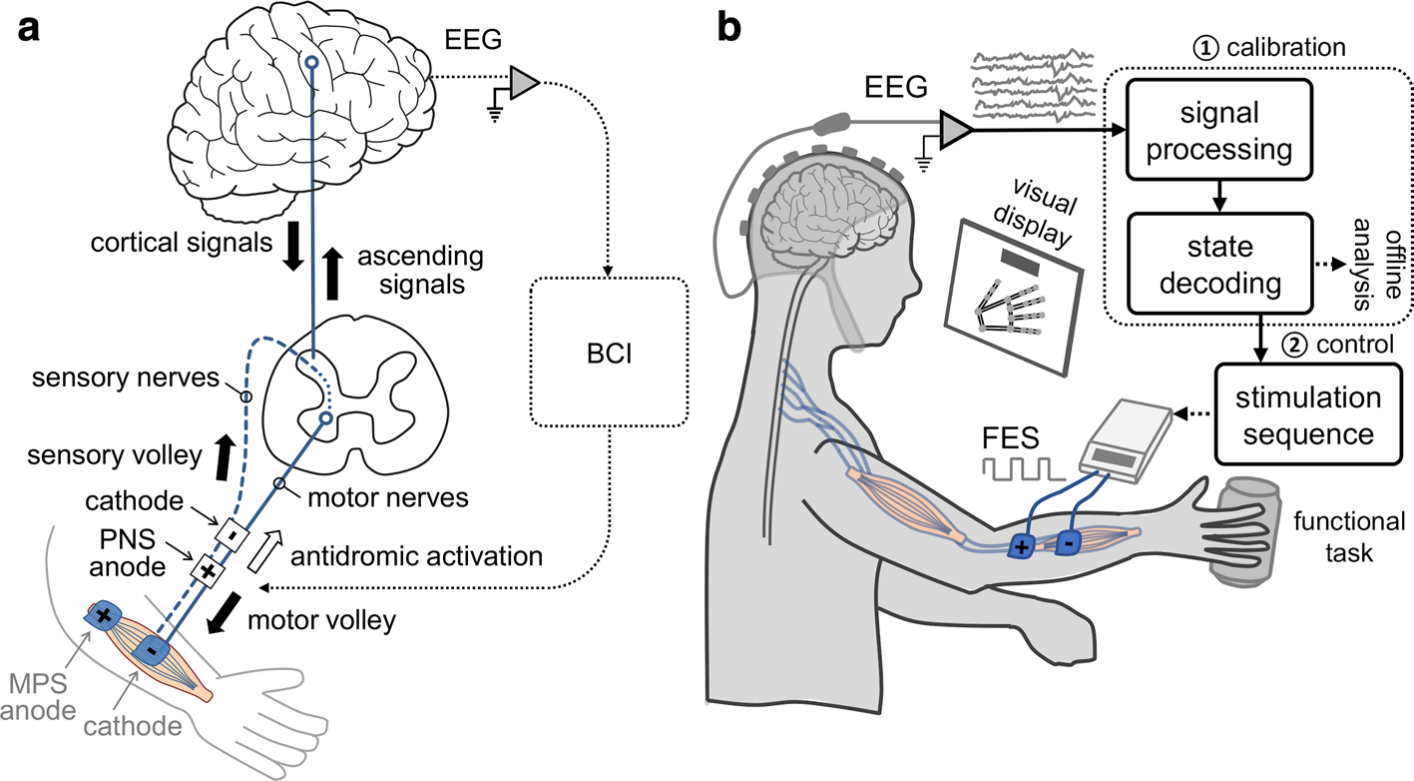
**a** Neurophysiological mechanisms of electrical stimulation of muscles and nerves—Illustration of the peripheral pathway generated via the efferent (motor) volley, and afferent (sensory) pathways, generated via the sensory volley during functional electrical stimulation (FES) of muscles and nerves. The antidromic activation along the motor axons and the sensory feedback traverses the spinal cord and activates the sensorimotor cortical networks to synapse with the cortical (descending) signals from the brain when a brain-computer interface (BCI) is used to trigger electrical stimulation. The figure shows electrode placement on the nerve trunk—peripheral nerve stimulation (PNS; black anode) and on the muscle belly—motor point stimulation (MPS; gray anode). **b** BCI activation of electrical stimulation—Illustration shows the components of the BCI system that can be used to trigger electrical stimulation of muscles and nerves via FES using non-invasive brain oscillatory recordings through electroencephalography (EEG). The main components include: (1) calibration of the state decoder (offline); and (2) control of FES system in real-time (online). During online control of FES, participants should perform functional tasks

Transcutaneous systems, which will be the focus of this article, are most frequently used for NMES, FES, and FEST to activate the motor system [119]. Electrical stimulators create a potential difference between two electrodes, a positive anode and a negative cathode, using surface (transcutaneous) stimulation electrodes [118]. The latest generation of FES systems are usually current regulated (compared to voltage regulated systems) as they can ensure that a fixed amount of charge is delivered to excitable tissue regardless of the impedance of the electrode-tissue interface. As illustrated in Fig. 1a, electrodes can be placed on: (i) the skin surface over the nerve trunk, which is referred to as peripheral nerve stimulation (PNS); or (ii) the belly of the targeted muscle, which referred to as motor point stimulation (MPS). During both PNS and MPS, stimulation of the peripheral nerves and muscle belly activate the mixed nerves (nerves that contain both motor and sensory fibers). Specifically, stimulation over the muscle belly activates nerves that contain both motor fibers and muscle spindle afferents. Moreover, nerve trunk stimulation targets the peripheral mixed nerves that contain and simultaneously activate both sensory (afferent) and motor (efferent) nerves. While H-reflex and M-wave recruitment patterns may differ between MPS and PNS [11, 100], in practice, during stimulation to produce functional movements, simultaneous efferent and afferent recruitment is expected. Moreover, muscles with the nerve trunks accessible for transcutaneous electrical stimulation are limited, e.g., trunk muscles cannot be activated via the nerve trunk stimulation [86]. In this case, muscle belly stimulation over the motor point is needed, which typically requires considerably higher stimulation amplitudes [8].

To generate muscle contraction, the impedance under the electrodes, as well as the location, size and orientation of the electrodes are important for optimizing the current [37]. Having a smaller cathode electrode and placing it close to the target nerve with the larger anode placed a distance away from the cathode can be used to generate more specific/accurate stimulation localization under the cathode while allowing a larger area of the skin under the anode to be used to close the electrical circuit and minimize discomfort under the cathode. Empirically, it is well-known that there are locations, where muscles are most sensitive to electrical stimulation, i.e., motor points (see [14] for upper limbs; [10] for upper body; and [21] for lower limbs). Larger muscles are known to have several motor points (e.g., seven quadriceps motor points were identified [21]), while smaller hand muscles typically have one optimal motor point [14]. Placement of electrodes on the motor point also plays an important role in generating strong muscle contractions [45].

### Physiological basis for generating muscle contractions using electrical stimulation

When a potential difference between the surface electrodes is created, the anode (“positive” terminal) attracts the negative and repels the positive ions towards the cathode (“negative” terminal), which attracts the positive and repels the negative ions. In effect, a current flow is created from the anode towards the cathode, which delivers an electric charge to the nerve trunk. At rest, the membrane potential of nerve cells and muscle fibers is approximately –70 to –90 mV with respect to the extracellular fluid. Applying electrical stimulation to the nerve trunk or muscle fibers can induce an electrical charge in the immediate vicinity of the outer membrane of the cell and change the rest membrane potential, which can in turn artificially elicit action potentials that can produce muscle contractions [8, 116]. While voluntary contraction induces action potential firing rate around 4–12 Hz [81], a higher stimulation frequency, around 20–50 Hz, is required to induce tetanic muscle contractions (see Sect. “Pulse frequency”).

When the nerve trunk is stimulated using electrical stimulation, both of the motor nerves (efferent nerves descending from the CNS to the muscles) and the sensory nerves (afferent nerves ascending from the sensory system to the CNS) are activated, as illustrated in Fig. 1a. Motor nerve activation generates direct contractions of the innervated muscles, while the sensory nerve activation can indirectly generate muscle contractions by eliciting the spinal reflex. Moreover, sensory stimulation, involving the reflex pathways, is thought to be beneficial for inducing neuroplasticity in the CNS [13, 41]. When electrical stimulation is applied over the muscle belly, the recruitment order is random, since activations depend on the distance between the electrodes and the nerve end terminals as well as the thickness of the nerve fibres [13]. Stimulation on the muscle belly activates localized muscle fibres around the electrodes, while nerve trunk stimulation activates muscle fibres evenly in the entire muscle belly [13]. Moreover, since muscle belly stimulation activates localized muscle fibres around the electrodes, repeated stimulation can also induce muscle fatigue [12, 81].

During electrical stimulation of muscles and nerves, electrical impulses propagate orthodromically along the motor axon towards the muscle to generate muscle contractions (motor volley in Fig. 1a). However, impulses can also propagate antidromically along the motor axon, away from the muscles towards the CNS (antidromic activation in Fig. 1a). This bidirectional propagation is unique to electrical stimulation and does not occur during voluntary activation of muscles. Antidromic activation along the motor nerves is generally considered to be a side effect of electrical stimulation. However, it has also been hypothesized that such antidromic propagation may play a role in neuroplasticity during electrical stimulation [126].

### Stimulation parameters

Different waveforms can be used to generate electrical impulses to stimulate the muscles. Galvanic (direct) current is not appropriate for generating neuromuscular contractions because it only produces an action potential at the moment it is turned on and off. More common are alternating currents waveforms, which deliver short electric impulses. Monophasic waveforms are disadvantageous as they could cause accumulation of electric charge in the tissue during prolonged electrical stimulation [8, 81]. Balanced biphasic impulses ensure that all residual charge left in the tissues is removed [8, 81]. However, such pulses generate contraction under both the anode and the cathode. Currently, sophisticated stimulation systems use asymmetric balanced biphasic impulses to ensure that the muscle contractions occur only under the cathode [81]. The magnitude of muscle contractions can be varied by changing the stimulating pulse amplitude (A), pulse width (PW) or the pulse frequency (f). Varying these parameters has been shown to have different neurophysiological effects during recruitment of motor and sensory pathways.

#### Pulse frequency

The action potentials in the CNS are frequency modulated, meaning that the intensity of the transmitted signal is proportional to the number of action potentials that occurs per unit time. Typical frequency of the nerve firing is around 4–12 Hz and the firing of the nerve fibres is asynchronous [81]. Depending on the application, a variety of frequencies can be used to generate contractions with FES. The most typical frequencies used in clinical applications range between 20 and 50 Hz [8]. These higher frequencies are needed, because electrical stimulation activates muscle fibres synchronously and as such requires higher firing rates to generate tetanic contractions [8]. Moreover, lower frequency stimulations (< 16 Hz) produce unfused contractions. They could also induce low-frequency fatigue, and they may not always be sufficient to elicit strong contractions [37]. On the other hand, high frequency stimulation (50-80 Hz) can induce rapid onset of muscle fatigue, which is a significant limitation of electrical stimulation systems [37]. However, higher frequencies of stimulation were reported to be more comfortable, because the response is smoothed [8]. Mang et al. [76] showed that high frequencies of peripheral stimulation at 100 Hz had larger central contributions, suggestive of having short-term neuromodulatory effects, compared to lower frequencies, which had no effect. Therefore, careful selection of stimulation frequencies can have a critical impact for inducing neurophysiological changes in the CNS during electrical stimulation. In our experiments, we typically used stimulation frequencies between 20 and 40 Hz for activation of upper-limb [58, 59, 62, 80, 88, 144], lower-limb [87, 147], as well as trunk muscles [86].

#### Pulse amplitude

The pulse amplitude, or the intensity, by which the stimulation is delivered is related to the depolarizing effect, with higher amplitudes inducing a stronger depolarizing effect. Typical FES pulse amplitudes rarely exceed 100 mA, while the exact levels depend on muscle properties, including the size of the muscle as well as the size of the stimulating electrodes and the pulse width of the simulating waveform. Smaller upper-limb muscles typically require smaller electrodes and lower pulse amplitudes to be contracted (e.g., 10–20 mA in [88]), while larger lower-limb and trunk muscles typically required larger amplitudes (e.g., 20–35 mA for contracting the soleus muscle in [87] and 20–25 mA for contracting the erector spinae muscle in [86]). Increasing the stimulation amplitude results in additional recruitment of smaller fibers near the electrode and larger fibers farther from the electrode [85]. With increasing amplitude, a threshold is reached beyond which no further fibers can be recruited, and no additional torque generated by the muscles. Moreover, very high intensities could lead to rapid muscle fatigue and discomfort during FES [8]. On the other hand, it was suggested that lower intensity stimulation (sensory stimulation) is more effective in inducing central changes in the CNS compared to higher intensities [13]. However, higher amplitudes of stimulation could be related to the increase in strength after FES training [37, 131].

#### Pulse width

Pulse width, or pulse duration, is the time span of a stimulating pulse. To achieve adequate depolarization of the nerve cells and cause muscles to contract, sufficient pulse width is required. Typical FES pulse width in clinical applications is between 200 and 500 μs. Short pulse durations (10–50 μs) have been shown to be selective in activation of muscle nerves, which can generate larger torque with a small number of muscle fibers [48]. However, very short pulse durations require larger pulse amplitudes to achieve adequate depolarization to contract the muscles. Larger pulse width was shown to produce stronger contractions [71], in addition to being able to penetrate deeper into subcutaneous tissue [23]. Longer pulse duration stimulation was found to be more effective for promoting central activation [5], likely due to activation of sensory axons [32, 33]. Preferential activation of motor axons using shorter pulse duration stimulation [48] and sensory axons using longer pulse duration [32, 33, 65] is probably related to the strength-duration constant of the sensory and motor axons [89, 146].

## Effects underlying electrical stimulation of muscles and nerves

Growing evidence suggests that FES can cause short- and long-term neurophysiological changes in the spinal and cortical neural circuits [26, 41]. Initially, FES was mostly employed as a permanent neuroprosthesis to regain function of paralyzed muscle (prosthetic use). It wasn’t until later that scientists seriously started to investigate the neurophysiological changes and gather evidence to show spinal and cortical re-organization after electrical stimulation. This evidence suggests that increased excitability can last anywhere from 5 min to several hours after a single session of electrical simulation [13], and for as long as several days after multiple sessions [83]. Moreover, recent results from FEST suggest that long-term clinical benefits could be obtained after use of electrical stimulation [59, 115, 116, 144]. Evidence also points out that therapeutic effectiveness is accompanied by long-lasting re-organization in the CNS [26, 88, 127, 132].

### Therapeutic effects

FES has been utilized as a prosthetic to restore various motor functions, such as standing (e.g., [1, 147]), sitting (e.g., [86, 145]), reaching and grasping (e.g., [2, 22, 96]), and more. In addition, it was also demonstrated that application of FES can have carry-over effects even after the stimulation was turned off. This phenomenon was first noticed by Merletti et al. [84]. Specifically, Merletti and colleagues observed that 2 months of stimulation of hand muscles using FES could improve the voluntary functional performance in some patients. Based on those findings, researchers started using FES as a therapeutic tool. Such interventions have been referred to as FEST [81, 114, 116]. Clinical improvements in reaching and grasping function after FEST was demonstrated in individuals with stroke and incomplete SCI [59, 79, 144]. However, despite clinical success of FEST, the exact mechanisms of the observed carryover effects are still not fully understood.

It is believed that the clinical changes after FEST could partially be due to the muscle training and strengthening as well as the improved flexibility and range of motion of the affected limbs [116]. In addition to the peripheral effects, it is thought that the spasticity reduction after FEST is one of the main reasons for clinical improvements in motor function [47, 62, 87]. However, compelling evidence of cortical brain re-organization after FEST has also been demonstrated recently [88, 132, 127, ]. It is hypothesized that the main mechanism behind the neuroplasticity induced by FEST, is the involvement of the voluntary intent during training [116]. During FEST, subjects are asked to attempt the movement, and only after their attempt, FES is applied on the muscles to assist movement completion. This idea was proposed by Popovic and colleagues [117]. Similarly, Rushton [126] suggested that the coincidence of the ascending and descending signals plays a significant role for enhancement of the synaptic connections. Specifically, during FES activation of muscles, antidromic impulses are also sent in the opposite direction along the motor axons towards the spine and the brain at the same time as the sensory volley is generated along the sensory axons (Fig. 1a). When the subject attempts the movement voluntarily, the descending motor commands are sent from the brain to the spinal motor neurons. It is thought that repetitive coincidence of these ascending (antidromic activation of motor axons and the sensory feedback) and descending signals over the course of multiple trials is responsible for the FEST-induced neuroplasticity. Next, we will discuss the neurophysiological effects of electrical stimulation of muscles and nerves, before returning to mechanism of associative neuroplasticity.

### Neurophysiological effects

#### Spinal reflex circuits

Evidence from studies examining the spinal reflex excitability (e.g., H-reflex), suggest that both short-term and long-term changes can be induced in the spinal circuits after application of electrical stimulation [13]. FES applied at intensities above the motor threshold generates tetanic muscle contractions via the efferent pathway, which may also antidromically activate the Renshaw cells [126] to inhibit spinal reflex excitability after the stimulation [53, 62, 87]. For instance, electrical stimulation was shown to reduce stretch reflex excitability in individuals with neurological deficits, which is beneficial for reduction of spasticity [47]. Specifically, as little as 60 s of FES applied over the soleus muscle, at intensities that evoked muscle contractions, can inhibit spinal reflex excitability in both the stimulated and non-stimulated muscles as well as in homologous and non-homologous contralateral muscles for at least 15 min after the intervention, while voluntary contraction-induced effects did not outlast the stimulation period and sensory-level stimulation did not affect spinal reflex excitability [87].

Prolonged application of electrical stimulation could also lead to long-term increase in reciprocal inhibition [105, 109], which may be beneficial for rehabilitation of individuals with CNS injuries. Moreover, in the upper-limb muscles, Kawashima et al. [62] showed a reduction in upper-limb spasticity indicated by the inhibition of H-reflex excitability after intensive upper-limb FEST intervention, which was accompanied by improvements in upper-limb motor outcomes. Unilateral electrical stimulation-induced contractions of upper-limb muscles can also affect spinal reflex excitability of homologous muscle pairs in the contralateral arm [53], as well as the lower-limbs bilaterally if the stimulation is combined with voluntary muscle contractions [60]. Therefore, electrical impulses that activate the mixed nerve bundle recruit not only the efferent motor axons to generate muscles contractions, but also the afferent sensory nerves via muscle stretch-induced feedback (muscle spindles) [13] or via antidromic propagation along the motor axons [126], which can affect both short-term and long-term spinal reflex excitability after the stimulating period.

#### Corticospinal tract

Electrical stimulation was also shown to affect the corticospinal excitability, which can be elicited using motor evoked potentials (MEPs) through single-pulse transcranial magnetic stimulation (TMS) applied over the primary motor cortex. A large body of literature has investigated how different modes of delivery and parameters of electrical stimulation can affect changes in corticospinal excitability (for a review, see [13, 26]). Increased MEP responses were observed following median nerve stimulation applied at the wrist when the stimulation was above, but not when it was just below, the motor threshold intensity [128]. Similarly, mesh glove stimulation, which targets widespread engagement of afferent fibers, was shown to increase corticospinal excitability for up to 1 h when it was applied at the sensory threshold intensity, but not at lower intensities [46]. Extent of corticospinal excitability modulation may also depend on the stimulation frequency. Using sensory level of stimulation, Mang et al. [76] showed that 100 Hz stimulation (high frequency) could increase corticospinal excitability, but they also reported that 200, 50, and 10 Hz stimulating frequencies were ineffective. When applied at motor threshold intensities, larger corticospinal modulation effects were obtained at higher frequencies, including 100 Hz and 20–50 Hz, compared to the 10 Hz stimulating frequency [56]. Generally, stimulation that can induce voluntary-like activations seem to be necessary to cause reliable changes in the CNS [31]. However, even at such intensities, no modulation of corticospinal excitability was observed immediately after each 45–60 min FEST session, while the cortical silent period was affected, implying short-term effects on cortical and/or subcortical inhibition [88] and involvement of sensorimotor integration [153]. It was recently shown that duration of FES delivery can also alter corticospinal modulation, with 20–40 min of stimulation facilitating MEP responses, while 60 min having no effects on corticospinal excitability [3], possibly due to effects of fatigue. Overall, the abovementioned studies point out that motor-level stimulation that can induce voluntary-like contractions, and stimulation delivered at higher frequencies that produce fused muscle contractions (not too high as to induce rapid muscle fatigue) are needed to evoke reliable excitability with the CNS.

Repetitive application of lower-limb electrical stimulation over multiple sessions was shown to increase corticospinal excitability after the stimulating period in the lower-limbs [143]. Moreover, increased corticospinal connections were shown following prolonged use of a drop-foot stimulator, which was accompanied with improvements in walking speed in people after stroke and multiple sclerosis [38]. In the upper-limbs, two hours of electrical stimulation was also successfully used to increase corticospinal excitability after the intervention, while aftereffects were relatively short-lived [122, 124]. However, longer-lasting changes in corticospinal excitability can be induced after approximately 40 h of FEST, with carryover effects outlasting the intervention in individuals with stroke [132, 127] and traumatic brain injury [88]. Overall, reinforcement of connections between the brain and the upper- and lower-limb muscles is generally associated with improvements in functional outcomes [13, 26, 134]. However, use of FES could also improve clinical outcomes, without any measurable changes in corticospinal excitability [9]. Overall, stimulation parameters have varied considerably between studies, which could significantly alter the neurophysiological effects [31, 42]. Similarly, effects of stimulation may be different between the upper- and lower-limb muscles [13], which could be due to their unique functional roles and different neural pathways involved in controlling these distinct segments. Nonetheless, careful selection of parameters is imperative for delivering effective neurophysiological changes both in the short-term during and after the stimulating period as well as to induce long-lasting sustained changes.

#### Cortical networks

Although electrical stimulation could affect excitability of both the spinal and the cortical networks, it is believed that short- and long-term excitability of the cortical sensorimotor networks can be engaged during and after electrical stimulation of muscles and nerves [26, 29, 41, 64, 122]. Specifically, 2 hours of electrical nerve stimulation applied at the wrist was shown to produce larger areas over which MEP responses can be evoked [124]. After traumatic brain injury, 3 months of FEST was required to elicit bigger MEP representations in the motor cortex, while cortical changes may also sustain after the intervention [88]. Motor maps obtained using TMS-evoked MEP responses can reliably extract somatotopic information from the primary motor cortex [148], providing evidence for cortical-level re-organization after application of FES [50].

This is further supported by recent evidence from neuroimaging studies showing that the state of motor cortical networks after application of FES is altered through multi-stage hierarchical processing which engages various parts of the motor system [6]. The somatosensory cortices, including both the primary (S1) and secondary (S2) somatosensory areas, are first activated during electrical stimulation of muscles and nerves [19, 26, 67, 103]. At intensities above the motor threshold, electrical stimulation activates cutaneous afferents as well as muscle spindles [35] to engage the S1 area [26, 151]. Activations in S2 appeared at lower intensities compared to S1 [7], while S1 activation was proportional to the intensity of stimulation [69]. Afferent recruitment via electrical stimulation also seems to have intensity-dependant modulation not only in the somatosensory cortex, but also in the primary motor cortical (M1) area, with larger activations at higher stimulating intensities compared to sensory-level stimulation [133]. As expected, cortical activation levels were larger during voluntary muscle contractions, compared to FES-induced movements in the M1, S1, and the supplementary motor area (SMA) [57]. However, S2 area activations were larger during FES-induced contractions, which may suggest possible direct activations [57].

Functional levels of stimulation, which can generate voluntary-like wrist flexion and extension, resulted in simultaneous cortical activations in the contralateral M1, S1 and premotor (PM) areas, bilateral S2 and SMA, as well as ipsilateral cerebellum activations [18]. Similarly, long-term FEST delivery induced widespread cortical re-organization characterized by increased contralateral cortical activations, as well as a similar trend in ipsilateral hemisphere activations, compared to pre-intervention assessments [88]. In stroke patients, 3 months of FEST resulted in either widespread activations distributed bilaterally in the somatosensory areas or more focused unilateral somatosensory activation after the intervention [127]. Similarly, FEST improved motor function in chronic stroke patients, which was accompanied by shifting in the somatosensory area activations from ipsilateral to contralateral hemisphere after the intervention [132]. Altered cortical activations in stroke patients after using FES were also shown in the lower-limbs with drop-foot stimulation, suggesting that SMA and angular gyrus regions play an important role in mediating carryover effects [43]. Moreover, short-term lower-limb FES application elicited significant activations of the sensorimotor networks (i.e., cerebellum and thalamus), with different neural activations achieved by adjusting the stimulation parameters [149]. Therefore, it seems that somatosensory cortex activations can be relayed to the motor cortical areas via cortico-cortical and/or cerebello-thalamo-cortical connections during electrical stimulation of muscles and nerves [26]. These results, therefore, suggest that peripheral electrical stimulation-induced activation of muscles can engage cortical sensorimotor networks in the widespread brain areas both during and after stimulation delivery.

## Brain-controlled electrical stimulation of muscles and nerves in rehabilitation

Now that we understand that electrical stimulation of muscles and nerves can engage the central nervous system, a question remains: how such changes can be utilized to maximize neuroplasticity? The answer may very well be related to associative stimulation and adjuvant techniques for electrical stimulation of muscles and nerves, specifically through brain-computer interface (BCI). During FEST delivery, participants are asked to actively attempt each movement and contraction before the therapist triggers the sequence of appropriate muscle activations using FES to assist task performance. Such task-specific and repeated training delivered with assistance of a therapist emphasizes the importance of associative interventions that combine activations at the cortical level and peripheral stimulation [88, 117] to induce experience-dependant cortical neuroplasticity [104]. This form of associative stimulation likely involves Hebbian plasticity [52], where a presynaptic input onto a postsynaptic neuron is strengthened as a consequence of simultaneous activation at the pre- and postsynaptic terminals [26]. Associative stimulation techniques that combine cortical and peripheral activations may include experimentally induced non-invasive brain stimulation, voluntary muscle contractions, motor imagery, and BCI control of electrical stimulation of muscles and nerves. Below, we will provide a brief overview of the neurophysiological mechanisms of these different forms of associative stimulation, while the main focus will remain on BCI-controlled FES.

### Associative stimulation of muscles and nerves

Adjuvant associative techniques that combine central activation at the level of the cerebral cortex and muscle contractions via electrical stimulation can be used to promote CNS neuroplasticity. Non-invasive brain stimulation can experimentally activate the cortical networks using various techniques. Specifically, paired associative stimulation (PAS) combines single-pulse transcranial magnetic stimulation (TMS) of the primary motor cortex (M1) and single-pulse electrical stimulation of the periphery to activate the afferent sensory circuits (for a review, see [27, 136]).

Using PAS, repeated cortical and peripheral stimuli, delivered at specific inter-stimulus intervals, can elicit long-term potentiation (LTP)- and long-term depression (LTD)-like plasticity using Hebbian rules of associative learning [136]. Specifically, it has been shown that mechanisms of PAS follow spike timing-dependant plasticity (STDP) (for a review, see [94]), where synaptic efficacy and polarity are determined by the temporal sequencing of pre- and postsynaptic terminal activity [24, 141]. Generally, facilitation of synaptic efficacy can occur if a presynaptic neuron fires before the postsynaptic neuron [40], while inhibition can occur if postsynaptic activations proceed or occur without presynaptic activation [16, 73]. Adherence to STDP rules during PAS was shown to be functionally relevant for increasing voluntary motor output [141], which can serve as a therapeutic tool to enhance recovery after injuries [24].

It has also been suggested that different forms of associative stimulation do not necessarily need to adhere to the STDP rules to achieve synaptic efficacy [26], with multiple possible pathways available to induce corticospinal neuroplasticity within the CNS [27]. For instance, when associative stimulation is applied with continuous trains of peripheral electrical stimulation, such as during FES, it is also possible to facilitate corticospinal excitability (e.g., [28, 123]). Similarly, repetitive TMS (rTMS) delivery of intermittent theta burst stimulation (iTBS) over the primary motor cortex can drive corticospinal excitability [54] and it was also shown to facilitate corticospinal excitability if applied before, but not after, electrical stimulation of the periphery [154]. Another form of non-invasive brain stimulation that could be effective for facilitating the cortical networks involves transcranial direct current stimulation (tDCS). When tDCS was applied over the contralateral M1 simultaneously as the medial nerve stimulation, corticospinal excitability facilitation was larger compared to when tDCS was applied alone, while electrical stimulation alone delivered with sham tDCS did not produce an effect [125]. Importance of associative form of stimulation is emphasized by the fact that stimulation of the periphery was generally less effective in facilitating corticospinal excitability when delivered alone. These findings show importance of brain state dependence for obtaining optimal effects when using non-invasive brain stimulation [55] and associative activation of muscles and nerves. Further evidence of cortical state dependency on corticospinal neuroplasticity has been illustrated through oscillatory beta cycle associative stimulation [63]. Details of non-invasive brain stimulation techniques are summarized in comprehensive reviews elsewhere (e.g., [55]). Overall, it is clear that multiple mechanisms likely determine the role of corticospinal neuroplasticity during associative stimulation, with modes of delivery of both cortical and peripheral stimulation playing a crucial function. While non-invasive brain stimulation techniques can undoubtedly offer important neurophysiological insights into associative stimulation mechanisms, which can be proven to be clinically relevant, the objective of the following section is to investigate how descending voluntarily commands can be synchronized with activation of peripheral activation of muscles and nerves.

Contractions that mimic voluntary-like movements were suggested to be important for generating cortical facilitation [26]. However, these effects are likely due to peripheral afferent feedback delivery through cutaneous and muscle spindle activation [151], which can also be applied by properly adjusting the stimulating parameters [13, 31]. While electrical stimulation alone offers a means of artificially producing muscle contractions by peripheral (direct) activation of the muscles without the central (cortical) drive, its effectiveness may be enhanced through voluntary contractions to a greater extent compared to electrical stimulation alone [35]. For instance, it was shown that delivery of electrical stimulation at the onset of muscle electromyographic (EMG) activity during wrist extension was successful in facilitating corticospinal MEP responses, while electrical stimulation alone was not [142]. Voluntary activations and electrical stimulation can also induce reciprocal changes in corticospinal excitability in agonist and antagonist muscles [155]. Using fMRI, the magnitude of cortical activation changes relative to rest were shown to be larger during voluntary contractions of upper-limb muscles compared to FES-induced movements in the M1, S1, and SMA areas [57]. However, combined voluntary and FES-induced contractions produced larger activations in the M1 and S1 areas compared to FES alone [57]. Although these studies suggest that voluntary activations can provide additional benefits compared to electrical stimulation of muscle and nerves alone, recent controlled trials investigating cyclic FES (i.e., without voluntary drive) and EMG-triggered FES (i.e., with voluntary drive) concluded that functional benefits may not be different between the two modes of delivery in stroke patients [90, 156]. On the practical level, individuals with neurological impairments may not always be able to generate sufficient or correct sequence of voluntary muscle contractions to reinforce electrical stimulation delivery.

Motor imagery can also offer a means to activate the cortical circuits during electrical stimulation of muscles and nerves. This strategy, in which patients imagine the precise execution of movements without any overt movement, is believed to engage similar neural networks as those involved in the actual production of movement [25]. It was shown recently that combined delivery of FES and motor imagery resulted in stronger cortical desynchronization compared to FES alone and motor imagery applied prior to delivery of FES [121]. When motor imagery was provided by means of watching and imagining actions shown on a pre-recorded video of grasping, while not producing the movements, it was shown that concurrent electrical stimulation facilitated MEP corticospinal excitability and that either motor imagery and electrical stimulation alone did not elicit any effects [157]. Similar acute effects were shown using combined motor imagery and electrical stimulation of the lower-limbs [137]. Preliminary results with chronic stroke patients also suggest that applying electrical stimulation in combination with motor imagery over the course of 10 days may possibly improve upper-limb function after the intervention cessation [106]. Tasks involving motor imagery and/or action observation can produce reliable and muscle-specific excitability of corticospinal responses in the upper-limbs (e.g., [39, 138]). Moreover, meta-analyses of a large body of literature investigating brain activations during motor imagery tasks concluded that the voluntary movements, action observation (visual display of tasks), and motor imagery alone (without the visual display) can all consistently give rise to activations in the premotor, parietal, and somatosensory cortical areas [51, 129]. Using motor imagery tasks, even without concurrent electrical stimulation, can therefore have numerous benefits in rehabilitation [135]. Considering associative stimulation, it is also of particular relevance that similar cortical areas activated by motor imagery are also recruited by electrical stimulation of muscles and nerves [26]. A practical consideration of motor imagery is that cortical activations are not necessarily ensured nor synchronized with the delivery of electrical stimulation. It is also well known that ability to produce motor imagery is subject-specific and that not all individuals can produce consistent brain activity with same effectiveness [77].

On the other hand, motor imagery can give rise to brain activity which can be detected using non-invasive brain activity recordings in real-time, i.e., electroencephalography (EEG). Such motor imagery-based phenomena typically include event related desynchronization (ERD) of EEG oscillatory cortical activity, which can be used in BCI applications to provide feedback to the users or control external devices [77, 138]. Single-trial movement-related cortical potentials (MRCP) is another movement-related EEG activity that can predict movement onset without actual motor activity [101, 102]. Regardless, of the method for producing movement-related brain activity (motor execution or imagination with and/or without visual cues), such approaches can be used to trigger a BCI system to control external devices. These serve as a basis for BCI-controlled FES systems, which can be used to activate electrical stimulation to ensure that cortical and peripheral stimulations are synchronized [91, 92]. A discussion about BCI-controlled FES associative stimulation follows. The primary focus of this work will be on upper-limbs, while there is existing an important body of literature examining lower-limb control using BCI associative stimulation (e.g., [91, 92]).

### BCI control for stimulation of muscles and nerves

Original BCI systems were developed to translate brain signals for the purpose of communication or control of artificial orthoses [152]. However, recent applications include replacing, restoring, enhancing, supplementing, or improving the natural outputs produced by the CNS [152]. Here, we will focus on the use of BCI systems to improve natural motor control through guiding activity-dependant plasticity that may be able to restore natural movements after neurological injuries. Use of BCI in rehabilitation for the purpose of improving motor function has gained considerable attention recently, with various applications summarized in comprehensive reviews elsewhere [20, 30, 72, 112]. For instance, a recent randomized trial used BCI to guide motor imagery during rehabilitation after stroke [113]. Compared to the control group, which performed motor imagery without feedback, the BCI group had greater functional gains after the therapy, suggesting that motor imagery-based feedback can also be used in rehabilitation [113]. Pairing motor commands from the BCI with the correct sequence of movements using a robotic orthosis can also result in cortical facilitation [49] and improved motor function after stroke [4, 120]. Specifically, a clinical trial also showed that using a BCI-controlled hand-arm orthosis immediately before the physical therapy session was more effective for improving upper-limb function compared to the control group which received randomly triggered orthosis before the therapy [120]. Similarly, using BCI-based robotic intervention was faster to improve upper-limb function after stroke compared to the control group which did not use the BCI system [4]. These studies agree that BCI can be used as a priming intervention to facilitate excitability of the sensorimotor cortical networks which can maximize the effects of subsequent therapy. Moreover, these studies suggest that that BCI systems can be used to facilitate associative Hebbian learning by pairing cortical activation with effective feedback using robotic orthosis or motor imagery to drive CNS neuroplasticity.

Considering the neurophysiological effects of electrical stimulation of muscle and nerve, which were presented in earlier part of this review (see Sect. “Neurophysiological effects”), BCI-controlled FES can also be viewed as a form of associative intervention that can be even more effective in facilitating feedback to the CNS to induce neuroplasticity and improve motor function. Indeed, in able-bodied people, BCI-FES systems were shown as more effective compared to motor imagery as feedback [15]. Use of BCI-FES was also shown to be effective for facilitating corticospinal excitability after short-term interventions [82]. A brief overview of the architecture of BCI-controlled FES systems will be presented next, followed by a review of clinical applications of BCI-FES for rehabilitation of upper-limb motor function as well as a summary of the proposed neurophysiological mechanisms of their action.

#### Architecture of BCI-FES systems

The proposed system architecture of typical BCI-controlled FES systems (e.g., [17, 34, 80,93, 97, 107]) is shown in Fig. 1b. Overall, BCI systems were mainly used as an EEG-trigger (i.e., brain switch) for activation of a pre-programmed FES neuroprosthesis. Almost all systems utilized a binary (one degree-of-freedom) control to detect: (i) rest; and (ii) active (movement) states. Although hand kinematic information [99] and even fingertip trajectories [98] can be decoded from cortical signals, these typically require intracranial electrodes and remain unfeasible for non-invasive BCI applications. Non-invasive brain recordings were typically obtained over the sensorimotor cortical areas using EEG signals, which were amplified and recorded through standard configuration procedures, with special precautions to avoid recording physiological or other artifacts (for a review of EEG signal acquisition, see [95]). During the acquisition stage, signals are typically band-pass filtered in the range of approximately 1 to 40 Hz, where a significant portion of the cortical oscillation signal power originates [110, 97, 95]. With little or no additional processing applied, signals are then recorded, and additional processing steps can be applied digitally.

Operation of the system is divided into two steps: (1) calibration of the state decoder (classification of rest or active states), which is performed offline; and (2) control of FES system in the real-time (online) (Fig. 1b). During the calibration stage, motor imagery-based tasks are presented using a visual display with the subjects relaxed and/or during motor execution attempt [34], while recording synchronous (or cue-based) EEG activity. The objective of this offline step is to select a combination of electrode sites and frequency bands that would be used for online control. The classifiers typically detect event related desynchronization (ERD) of brain oscillatory activity [58], which is typically present before motor tasks [44]. During rest, brain activity in the sensorimotor areas can be characterized by synchronous oscillatory patterns, while prior to the movement onset or before attempted or imagined movements, desynchronization (ERD) of specific frequency bands occurs [111]. Overall, ERD can be a reliable biomarker for detecting motor cortical activity using EEG recordings, and it has been shown to reflect excitability of the primary motor cortex [139] and spinal motoneurons [140]. Considering that ERD frequency characteristics are subject-specific [111], a common procedure is to plot the time–frequency signal power to help manually identify the ERD frequency bands that will be used for online control for each participant (e.g., [34, 58, 80, 97]). This emphasized the necessity of the calibration step, while re-calibration is commonly required prior to each training session. Alternatively, signals can be subdivided into typical frequency bands: (a) alpha band (8–13 Hz), also know as mu band in the sensorimotor area, which is typically associated with restfulness states; and (b) beta band (13–30 Hz), which is associated with various active concentration tasks, attention, or excitement. While frequency bands can be adjusted based on various neurobiological considerations [95], typically mu and beta bands were used in most BCI-FES applications. The power of these pre-determined frequency bands or other spatiotemporal features of the signal can then be fed into a multi-feature linear discriminant analysis (LDA) classifier [93, 107] or other machine learning-based methods [17, 74]. In case of machine learning, the signals are typically log transformed to normalize the data [107]. Similarly, electrode location(s) that will be used for online control of the BCI system can be selected manually from the most discriminant (e.g., [34]) or partially from a set of relevant candidate location and fed into an LDA classifier (e.g., [93]). This can result in several locations [93] or a single EEG channel [58, 80] used as a BCI control signal. An alternative, approach is to use spatial filtering technique instead of manual selection for EEG source localization. Such spatial filters in BCI applications includes common spatial pattern (CSP), large Laplacian spatial filter (LLSF) and optimized spatial filter (OSF) [101, 102]. Spatial filters have been applied in BCI-controlled FES systems [17]. However, manual selection of features was always considered to account for discriminatory and prior neurobiological knowledge of the features used for BCI control [17, 58, 80, 93]. Once the feature space is selected, a set of “rules”, in terms of discriminant power of the EEG signals recordings, is determined based on the machine learning algorithm [17, 93, 107] or as a simple signal power threshold (e.g., [34]). Typically, in the online operation mode, these rules can be updated automatically based on a running average performance [34, 107] or by the experimenter [80] to adjust for the slow signal drifting. Thus, BCI-FES systems operation depends on the effective collaboration of two adaptive controllers, the BCI system and the brain [152], which may prove to be critical for inducing neuroplasticity.

To control the FES neuroprosthetic in real-time, the EEG-trigger brain switch can be operated using asynchronous (non-cued) mode or simple synchronous (e.g., GO cue) configuration using a visual display [96]. Typically, the user attempts a movement over a period of time, while the algorithm detects the state of the decoder (e.g., rest or active) in a fixed timeframe, after which FES delivers a corresponding pre-programmed sequence of muscle activations. If the algorithm can not detect a change in the state, either the experimenter can provide manual control (e.g., [58, 80]) or the trial be considered a “no decision” [17]. In addition to physiological and other artifacts, an inherent problem of BCI-controlled FES systems is that the recorded EEG activity is noisy during activation of FES. Several artifact reduction signal processing techniques have been compared with an intracortical BCI-FES system [158]. Linear regression referencing (LRR), which creates channel-specific reference comprised of the weighted sums of other channels by assuming that artifact is similar across channels, was shown as superior compared to other method such as common average referencing and blanking methods [158]. However, most non-invasive systems in rehabilitation utilize a brain switch BCI, whereby EEG recording is turned off after the decoding, while FES is applied. This can limit the applications of non-invasive BCI-FES to synchronous (cue-based) operation. While non-cued (asynchronous) BCI are desirable for prosthetic applications to restore natural function, rehabilitation applications aim to improve voluntary motor function through BCI-FES training. Specifically, the goal of BCI-FES rehabilitation is to facilitate associative Hebbian learning by pairing cortical activation with FES, which can effectively be accomplished using cue-based BCI operation. Overall, complexity of the BCI-FES system should be balanced to consider accuracy as well as practical considerations required for clinical implementation. These considerations present some general system architecture of the existing BCI-FES systems.

#### Examples of BCI-FES for restoration of upper-limb motor function

A summary of the reviewed non-invasive BCI-controlled FES literature in the field of motor rehabilitation is presented in Table 1. Most clinical applications of BCI-FES in rehabilitation has been performed in stroke patients and a majority of these are single-subject case studies [34, 58, 80, 93]. This body of literature has provided vital data regarding feasibility of clinical implementation and hypotheses related to mechanisms of recovery. Notably, Daly and colleagues [34] used a BCI-system to control FES to control voluntary finger function in an individual who lost voluntary upper-limb control as a result of a stroke sustained 10 months prior to the study. Using an ERD signal power threshold-based method to detect a change of the beta frequency band oscillations in the sensorimotor area, the BCI system triggered finger extension and rest states via FES in the contralateral hand. After only nine sessions, the participant’s ability to produce individual finger movements voluntarily was improved [34]. Moreover, in a series of case studies, Marquez-Chin and colleagues [58, 80] showed that a single channel power change either in the beta [80] or mu oscillations [58] recorded over the sensorimotor area (note: specific location was adjusted during calibration for each participant) could be used as a threshold-based EEG-trigger to control FES effectively for facilitating reaching and grasping. Although most BCI-FES studies were used to generate single joint movements, functional task performance during therapy [80], as shown in Fig. 1b, is relevant to induce activity-dependant plasticity. Using such a system, they showed that 40 one-hour sessions induced meaningful clinical improvements indicating upper-limb recovery and functional independence improvements [58, 80]. Another clinical case study with a hemiplegia patient by [93] used a BCI system to detect finger extension or rest states through an LDA classifier that was utilized to discriminate ERD power changes of a 4-dimensional feature space recorded from EEG data (mu and beta frequency bands of left and right hemisphere sensorimotor areas). In a single-subject crossover design, the results of their study showed that BCI-FES use lead to marked lateralization of cortical activations [93], compared to the initial assessments which indicated diffuse fMRI activations of sensorimotor area. Moreover, changes in corticomuscular coherence were also shown in addition to the clinical improvements in the upper-limb function. Taken together, these studies present feasibility for clinical application of BCI-FES therapy (BCI-FEST) for improving motor function, which could also be related to changes in the state of cortical sensorimotor networks.

**Table 1:** Summary of non-invasive BCI-controlled FES studies used for rehabilitation of upper-limb motor function

Study	Population	BCI	FES	Intervention	Main results
Daly et al. [34]	1 stroke patient (F, 43 y)	1-channel EEG trigger detected by signal power change using a threshold method to classify rest vs. active states	1-channel FES applied to facilitate finger extension movements	Case study intervention: 9 session of 45 min delivered 3 times per week for 3 weeks	Participant’s ability to produce voluntary finger movements was improved after 9 sessions
Mukaino et al. [93]	1 stroke patient (M, 38 y)	Multi-channel EEG trigger using an LDA classifier detected signal power change of a multi-feature space to classify rest vs. active states	1-channel FES applied to facilitate finger extension movements	Case study crossover control design: (i) BCI-FES or (ii) FES was delivered for 60 min over the course of 2 weeks (10 days in total)	Clinical improvements and muscle tone changes were seen after BCI-FES as well as lateralization of cortical activations and affected corticomuscular coherence
Li et al. [74]	15 stroke patients (BCI-FES: n = 8, 5 M/3 F, 67.0 ± 5.0 y; control: n = 7, 6 M/1 F, 67.1 ± 6.0 y)	Multi-channel EEG trigger using an SVM classifier was used to detect rest vs. active states	1-channel FES applied to facilitate wrist extension movements	Randomized controlled intervention: (i) BCI-FES or (ii) FES training was delivered three times per week for 8 weeks	Improvements in motor function, activation of bilateral hemispheres, and altered activation of the sensorimotor cortexes was shown after BCI-FES intervention.
Kim et al. [66]	30 stroke patients (BCI-FES: n = 15, 6 M/9F; 59.1 ± 8.1 y; control: n = 15, 6 M/9F, 59.9 ± 9.8 y)	2-channel EEG trigger detected attention-related sensory motor rhythm using a threshold to classify rest vs. active states	1-channel FES was applied to stimulate wrist extensor muscles of the affected upper-limb	Randomized controlled intervention: (i) BCI-FES or (ii) conventional therapy (control) was delivered for 30 min per session over 4 weeks	Improvements in functional mobility and range of motion, suggesting improved motor function, was shown after BCI-FES intervention.
Marquez-Chin et al. [80]	1 stroke patient (M, 64 y)	1-channel EEG trigger detected by signal power change using a threshold to classify rest vs. active states	Multi-channel FES facilitated reaching movements: (i) forward reaching/retrieving (ii) reaching to the mouth, and (iii) lateral reaching	Case study intervention: 40 sessions of 90 min of BCI-FES were delivered 3 times per week	Improvements in clinical scores as well as the changes in arm function were shown after 40 sessions.
Osuagwu et al. [107]	12 SCI patients (BCI-FES: n = 7; FES: n = 5; 12 M, 51.7 ± 18.4 y)	Multi-channel EEG trigger using an LDA classifier detected signal power changes of a feature space to classify rest vs. active states	Multi-channel FES was applied to facilitate hand extension or flexion of both hands during active states	Randomized controlled intervention: (i) BCI-FES or (ii) FES were delivered 3–5 times weekly for 1 h (20 sessions in total)	BCI-FES therapy results in better neurological recovery and improvements in muscle strength compared to FES
Biasiucci et al. [17]	27 stroke patients (BCI-FES: n = 14, 6 M/8F, 56.4 ± 9.9 y; control: n = 13, 10 M/3F, 59.0 ± 12.4 y)	Multi-channel EEG trigger using a Gaussian classifier was used to discriminate rest vs. hand extension states	1-channel FES was applied to facilitate hand extension movements	Randomized controlled intervention: (i) BCI-FES or (ii) FES were delivered two times per week for a period of 5 weeks (10 sessions in total)	Improvements in motor function were accompanied by increase in functional connectivity between motor areas in the affected hemisphere after BCI-FES
Jovanovic et al. [58]	1 stroke patient (M, 57 y)	1-channel EEG trigger detected by signal power change using a threshold to classify rest vs. active states	Multi-channel FES facilitated functional movements: (i) hand opening/closing, and (ii) arm reaching/retrieving (varied between sessions)	Case study intervention: Two 40 one-hour BCI-FES sessions (80 sessions in total) were delivered with 3 sessions per week	Improvements in clinical scores and functional capacity were shown after completion of 80 therapy sessions

*BCI* Brain-computer interface, *EEG* Electroencephalography, *FES* Functional electrical stimulation, *LDA* Linear discriminant analysis, *SCI* Spinal cord injury, *SVM* Support vector machine

Only recently larger studies using BCI-FES training with stroke patients have been reported in the literature [17, 66, 74]. Specifically, in a randomized controlled trail, Kim et al. [66] showed greater functional improvements using BCI-FES compared to FES training alone. The authors presented evidence that training with BCI-FES five times per week during a 4-week period could improve clinical scores associated with upper-limb functional recovery, while they did not present evidence to explain the possible mechanisms or recovery. Another smaller trial with stroke patients by Li et al. [74] also showed better functional recovery using BCI-FES, compared to the use of FES alone, for upper-limb rehabilitation after stroke. Specifically, their results showed motor functional improvements after 8 weeks of BCI-FES training, which was also accompanied by activation of bilateral cerebral hemispheres, while activation of the affected sensorimotor cortex and parietal lobe were suggested to contribute to function recovery [74]. The most comprehensive clinical trial in stroke patients thus far was performed by Biasiucci and colleagues [17]. In a clinical study with 27 chronic stroke survivors, participants were divided into two groups to compare BCI-controlled FES and FES alone interventions using otherwise comparable modes and intensities of delivery for stimulation of muscles. The BCI-controller was developed using a machine learning approach with a number of physiologically relevant features recorded from EEG signals over the sensorimotor areas of the affected hemisphere to detect the rest and active states targeting extension of the affected hand. The intervention was applied two times per week for a period of 5 weeks, while assessments were performed before and after the intervention as well as in follow-up after the intervention period. The results of this trial showed that BCI-controlled FES group exhibited clinically relevant and longer-lasting functional recovery results compared to the FES group. Specifically, the BCI-group exhibited functional recovery profiles which lasted 6–12 months after the invention, while increased functional connectivity between motor areas during voluntary hand contractions in the affected hemisphere were correlated to functional improvements [17]. Together, clinical trials in stroke patients present evidence that functional motor improvements are associated with enhanced cortical activations in the affected hemisphere. Moreover, they all agree that BCI-controlled FES is more effective in producing functional and cortical changes compared to FES delivery alone.

Fewer BCI-FES applications have been implemented in individuals with SCI [78, 96, 97, 107, 110]. Importantly, control of BCI and FES has been demonstrated in individuals with tetraplegia [110]. Most early applications of BCI were utilized to control an implanted upper-limb FES neuroprosthesis in patients with complete SCI [96, 97]. Other detailed reports showed the efficacy of BCI with intracranial electrodes to control FES [78]. These studies paved the way to the current research which uses these two technologies as a therapeutic intervention. Specifically, a recent study by Osuagwu and colleagues [107] applied BCI-FES as a rehabilitation intervention in twelve subacute tetraplegic patients with incomplete injuries (C4-C7; ASIA B/C) who were subdivided into BCI-FES and FES groups. The BCI controller was implemented to detect desynchronization (ERD) of beta frequency EEG cortical oscillations using an LDA classifier to discriminate between active and rests states. In the BCI-controlled group, FES was used to apply a sequence of hand extension and flexion tasks during the active state or remain at rest in the rest state. The control intervention group received an equivalent open-loop controlled FES (10 s ON/10 s OFF). Range of motion was improved in both groups, while muscle strength was observed in the BCI group only after the intervention, compared to the pre-intervention assessments, to suggest functional improvements. Initial assessments revealed that cortical desynchronization (ERD) during movement attempt was not focused in the sensorimotor area in both groups, while 20 sessions of BCI-FES intervention resulted in more focused cortical EEG activity and remained widespread in the FES group [107]. It is relevant to point out that the electrode locations for controlling the BCI-FES system were located over the sensorimotor cortices. Therefore, use of BCI-FES may be able to promote re-organization after incomplete SCI by focusing associative activations to a specific cortical area through a BCI system.

#### Proposed mechanism of associative motor learning using BCI-FES

Overall, most of the literature in stroke and incomplete SCI individual showed evidence for improved motor function after using BCI-controlled FES system, compared to the control conditions which usually involved FES delivery alone (e.g., [17, 107]). Evidence from these studies also suggests that cortical level re-organization is correlated to functional recovery benefits. Specifically, results in stroke patients [17, 74, 93, 107] suggest that improved sensorimotor activations in the affected hemisphere may be related to functional improvements. Intact motor areas topologically adjacent to the damaged site within the primary motor cortex (M1) and other sensorimotor areas such as the premotor cortex (PM) and supplementary motor areas (SMA) in contralateral and ipsilateral hemisphere may assume control over the affected muscles via intricate intracortical connectivity networks after brain injury [104, 130, 150]. More focalized sensorimotor cortical activations were also reported after BCI-FES interventions in people with incomplete SCI [107]. BCI system can translate brain signals into a novel type of output [152]. Through such use- or activity-dependant associative stimulation, BCI-controlled FES interventions may create new pathways for generating and transmitting neuronal commands from the cortex to the muscles of interest.

So, how can BCI-controlled FES be used to effectively induce neuroplasticity in the CNS? The likely mechanism for neurological basis for rehabilitation is that BCI can provide a way to modify neuronal activity with progressive practice that includes feedback and reward [36]. Facilitation of motor recovery through error-based or reward-based learning most probably involves Hebbian-like plasticity [52], where a presynaptic input onto a postsynaptic neuron is strengthened as a consequence of simultaneous activation at the pre- and postsynaptic terminals. Cortical oscillatory desynchronization (ERD), which has primarily been used as a trigger for BCI-controlled FES, was shown to reflect excitability of the motor cortical [139] and spinal motoneuronal [140] networks. Similarly, electrical stimulation of muscles and nerves has been shown to activate cortical (e.g., [18]) and spinal motor networks (e.g., [53, 60]) during the stimulation. Therefore, a presynaptic input in the form of oscillatory cortical desynchronization which is detected by the BCI system can generate strengthened connections due to simultaneous postsynaptic activation using FES activations of similar sensorimotor networks. The candidate mechanism of such reward-based learning within the brain is probably upregulation of dopaminergic excitatory receptors and/or downregulation of GABAergic inhibitory receptors [104]. Adherence to STDP rules can be relevant for increasing voluntary motor output to enhance therapeutic outcomes [24,141]. However, it has also been suggested that associative stimulation may not need to follow the strict timing principles [26]. As indicated by the studies using rTMS [154] or BCI [113] to pre-activate (or prime) the cortical sensorimotor networks before delivery of therapy, activations should at least remain within some reasonable associative timeframe, while gains may be maximized by optimizing delays between pre- and post-activations.

Evidence of adaptive cortical re-organization also exists. Specifically, more focal cortical activations were observed after training using BCI-controlled systems that utilized the same sensorimotor areas to control FES during the intervention [17, 107]. Although prior neurobiological considerations were taken in most applications, electrode locations for controlling the BCI were chosen (either manually or through machine learning) to include cortical areas that produced best discriminatory selectivity to maximize accuracy of the controller [17, 58, 80, 93]. Similarly, classifier decoder was typically adaptively adjusted during operation to optimize performance [34, 80, 107]. Therefore, to achieve high reliability and accuracy, the system adapts to the user behaviours. However, it must be kept in mind that BCI control requires constant modification of two adaptive controllers, i.e., the decoding system as well as the brain [152]. A recent elegant study in non-human primates, showed that BCI-controlled FES can be used to induce adaptive cortical changes throughout different sensorimotor cortical sites [61]. Specifically, through use of a BCI system, cortical activity became localized around an arbitrarily selected cortical site that was used for controlling FES of upper-limb muscles in primates. The targeted cortical areas, which included locations in the primary motor (M1), premotor (PM), and somatosensory (S1) cortex, could be reset and localized to a new site rapidly using BCI-FES training [61]. Although the evidence was shown in non-human primates using invasive techniques, this study provides important implications that BCI-FES system should balance adaptive control to guide neuroplasticity within specific cortical areas. Constant modification of two adaptive controllers can, therefore, enhance CNS recovery.

#### Future trends in non-invasive BCI-controlled FES rehabilitation

Current evidence is in support of using non-invasive BCI-controlled FES for improving upper-limb motor function. Indeed, when FES systems were coupled with BCI, the observed outcomes and cortical facilitation seemed to outperform FES alone after stroke [17] and SCI [107]. Nonetheless, numerous issues remain to be resolved in the future. Most current non-invasive BCI-FES applications use one degree-of-freedom control to detect rest and active states, which can generate simple movements such as wrist or finger extension (Table 1). Such goal based BCI operation that uses EEG as a trigger to activate a pre-programmed FES sequence may limit full potentials of this technology, despite the promising results so far. Future non-invasive BCI-controlled FES systems should aim for continuous control of functional tasks which involve several muscles such as during FEST interventions (e.g., reaching and grasping an object). This has recently been demonstrated using in an individual with SCI using an implanted upper-limb FES neuroprosthesis and intracortical electrodes [2]. Continuous control and decoding may also be advantageous in capturing rapid cortical networks dynamics, which can be implemented in adaptive control. For non-invasive systems, this will only become possible with the advancements in sensing technologies as well as improvements in neural decoding through use of machine learning or other algorithms capable of capturing complex cortical dynamics. The issue of FES artifact removal from EEG recordings will also have to be resolved in the future before continuous and asynchronous (non-cued) BCI-FES operation can be realized. In parallel, as the technology develops, understanding the underlying cortical re-organization both during BCI-FES operation and its interventional potentials through clinical trials will also be critical for engineering neuroplasticity.

## Conclusions

Electrical stimulation of muscles and nerves can be used to generate muscle contractions and create functional movements of limbs. The implication of the motor and sensory recruitment of muscles with electrical stimulation goes beyond simple contraction of muscles and creation of functional movements. There is growing evidence to suggest that electrical impulses, which traverse the spinal cord and ascend to the brain, can induce short- and long-term neurophysiological changes in the CNS. These changes are likely responsible for the therapeutic effects that have been demonstrated in clinical studies using FES therapy (FEST). Most clinical applications have focused on generating functional contractions. However, involvement of the sensory afferent information during electrical stimulation is thought to be critical for modulating the CNS circuits. Stimulation parameters, such as pulse amplitude and pulse duration, determine which neural fibers will be recruited, and the frequency of stimulating wave determines the rate at which the action potentials are depolarized. Recruitment of muscles and nerves using such stimulation can facilitate excitability of spinal reflex circuits and cortical networks. Moreover, long-lasting changes in the CNS may be enhanced by synchronization of cortical and peripheral activations through associative stimulation. Brain-controlled technologies offer a way to synchronize descending cortical commands and successful execution of the intended tasks using a FES, which can promote associative Hebbian learning. Emerging clinical evidence indeed suggests that BCI-controlled FES is an effective rehabilitation intervention that can possibly outperform FES alone. Future BCI-FES applications should aim to achieve continuous and functional task control (BCI-FEST) while adaptively modifying the control dynamics based on underlying cortical re-organization to engineer neuroplasticity in the CNS and maximize recovery of motor function in individuals with neurological injuries.

## Data Availability

Data sharing not applicable to this article as no datasets were generated or analysed during the current study.

## Abbreviations

BCI: Brain-computer interface
CNS: Central nervous system
CSP: Common spatial pattern
EEG: Electroencephalography
EMG: Electromyography
ERD: Event-related desynchronization
FES: Functional electrical stimulation
FEST: Functional electrical stimulation therapy
fMRI: Functional MRI
LDA: Linear discriminant analysis
LLSF: Large Laplacian spatial filter
LRR: Linear regression reference
LTD: Long-term depression
LTP: Long-term potentiation
M1: Primary motor cortex
MEP: Motor evoked potentials
MPS: Motor point stimulation
MRCP: Movement-related cortical potentials
MRI: Magnetic resonance imaging
NMES: Neuromuscular electrical stimulation
OSF: Optimized spatial filter
PAS: Paired associative stimulation
PM: Premotor area
PNS: Peripheral nerve stimulation
PR: Parietal area
tDCS: Transcranial direct current stimulation
TMS: Transcranial magnetic stimulation
rTMS: Repetitive TMS
S1: Primary somatosensory area
S2: Secondary somatosensory area
SCI: Spinal cord injury
SMA: Supplementary motor area
STDP: Spike timing-dependant plasticity
SVM: Support vector machine
